# Bridging regulation and practice: CJEU and Dutch case law on botanical health claims

**DOI:** 10.3389/fphar.2025.1523904

**Published:** 2025-02-19

**Authors:** Karin G. M. Lenssen, Alie de Boer

**Affiliations:** ^1^ Food Claims Research Centre, Maastricht University, Venlo, Netherlands; ^2^ University College Venlo, Maastricht University, Venlo, Netherlands

**Keywords:** botanicals, European food law, food information, voluntary food information, risk analysis

## Abstract

**Introduction:**

Even though botanicals are increasingly popular ingredients for food supplements, health claims related to their putative bene ts remain unclearly regulated.

**Methods:**

Through an analysis of EU and national case law from the Netherlands, including self-regulatory decision-making, we have studied the implications of case law on botanical health claims.

**Results:**

Our analysis reveals that there are multiple issues related to claims on botanical-containing products: whether it should be classi ed as food or medicine; what statements should be understood as health claims; what type of evidence should underlie health claims and, more specically, botanical health claims; and how to deal with online commercial communication. The case law analysis highlights rst that a gray area will continue to exist when classifying products as foods or medicinal products, particularly when it comes to products that contain botanical ingredients. Most importantly, our study also reveals that claims—even when they are on hold, like botanical claims—need a certain scienti c foundation before they can be used on products. Finally, the courts believe that even though on-hold claims will continue to give a certain level of uncertainty for food business operators, this is not a legal but rather a regulatory issue.

**Discussion:**

The findings from our case law analysis highlight that even though case law is useful in further interpretation of legislation, it does not provide any policy advancement. In the case of botanicals, a political decision regarding their substantiation is highly desired.

## 1 Introduction

The use of herbal or botanical food supplements is widespread in the European Union (EU) (Garcia-Alvarez et al., 2014; Papatesta et al., 2023). These products, made from one or more botanical species and sold in dosed form, are used by consumers for their alleged beneficial effects on health (Kloosterman et al., 2022). Botanicals are defined as products derived from plants, algae, fungi, or lichens (EFSA, 2018). The use and sales of botanical food supplements have been increasing for years and are expected to continue to grow (Euromonitor International, 2018).

Botanical supplements are classified and regulated as food products in the EU. The communication of health benefits of these products is regulated by Regulation (EC) No 1924/2006 on Nutrition and Health Claims (NHCR), which requires that health claims are substantiated with generally accepted or newly developed scientific evidence (European Parliament and the Council of the European Union, 2006). After being subjected to an authorization procedure in which the underlying scientific evidence is reviewed by the European Food Safety Authority (EFSA), health claims may be authorized for use on food products by the European Commission (Commission). An ongoing debate on the substantiation of botanical health claims prevents the full implementation of the NHCR for the health claims on botanicals (Lenssen et al., 2020). The authorization procedure for these botanical health claims is on hold: the proposed health claims on botanicals have not yet been subjected to the full authorization procedure. Until the procedure is completed, the botanical health claims are subject to transitional measures, as described in Art. 28.5 of the NHCR (European Parliament and the Council of the European Union, 2006). In its check of the regulatory fitness of the NHCR, the Commission concluded that, although the NHCR is still relevant, the current situation regarding botanical health claims has a negative impact on food business operators (FBOs) and consumer protection (European Commission, 2020).

The authorization procedure of the botanical health claims has been on hold for more than 10 years and is not expected to be resolved soon. The inconclusive and unclear status of botanical health claims has affected the work of enforcement authorities, resulting in national court cases on the potential violation of relevant provisions in the NHCR. For the implementation of EU regulations in national courts, the Court of Justice of the European Union (CJEU) can be requested to provide preliminary rulings, which have happened in various instances. As a result, these CJEU rulings have significantly shaped the implementation of the NHCR, especially in relation to the application of the transitional measures. The main aim of this study is to understand how CJEU case law on health claims and botanicals shaped the development of the NHCR and, more specifically, these transitional measures. To study this question, the interpretation of the NHCR in CJEU cases was reviewed. To further understand the implications of this EU case law, the practical translation of the CJEU cases in Dutch national court cases and Dutch advertisement committee cases were analyzed. Together, this review shows the implications of CJEU cases issued because of the on-hold status of the botanical health claims and how these impacted the interpretation of the NHCR.

## 2 Regulatory and conceptual framework

Botanical supplements are regulated as food supplements, making them subject to the regulatory framework of food products (European Parliament and the Council of the European Union, 2002a). The basis of EU food law can be found in Regulation (EC) No178/2002, also known as the general food law (GFL) (European Parliament and the Council of the European Union, 2002b). The general aim of this regulatory framework for foods is to ensure the effective functioning of the internal market and to protect consumers (European Parliament and the Council of the European Union, 2002b). The NHCR, implemented to regulate communication on the health benefits of food products, defines information on nutritional value and health attributes of food products as nutrition and health claims. Such claims must be substantiated by generally accepted scientific evidence (Art. 6), which is evaluated in the pre-market authorization procedure (European Parliament and the Council of the European Union, 2006). The evaluation constitutes an assessment of the scientific evidence compiled in a scientific dossier by EFSA (European Parliament and the Council of the European Union, 2006). EFSA evaluates the evidence in three distinct steps: (i) the bioactive substance must be sufficiently characterized, (ii) the claimed effect must be a beneficial physiological effect, and (iii) there must be a cause-and-effect relationship between the bioactive substance and the beneficial physiological effect (de Boer et al., 2014; EFSA NDA Panel et al., 2017; van Steenwijk et al., 2021). The evaluation of the criteria is published in a Scientific Opinion. After weighing the outcome of the assessment and other relevant considerations, the Commission decides upon the authorization of a health claim.

### 2.1 Health claims on botanicals

Upon implementation of the NHCR in 2006, FBOs could submit the scientific dossiers for putative general function health claims, article 13.1 claims, until 31 January 2008, after which EFSA was asked to evaluate these dossiers (European Parliament and the Council of the European Union, 2006). Many claims, including several reviewed on botanicals, received a negative opinion from EFSA’s Panel on Nutrition, Novel Foods and Food Allergens (NDA Panel) and were subsequently not authorized (European Commission, 2020). Quickly thereafter, discussions commenced on the comparability of botanicals in food and medicinal products and the seemingly stricter review process for the health benefits of foods, for which different assessment criteria apply (Lenssen et al., 2020).

The EU regulatory frameworks for food and medicinal products are mutually exclusive: a product is categorized as either a food or a medicinal product (Melcher and Timmermans, 2009). Any statement that refers to the treatment, cure, or prevention of a disease would classify a product as a medicinal product (Verma, 2013). For food products, including botanicals and botanical supplements, health effects must be substantiated with scientific studies, including blinded, randomized human intervention trials (EFSA NDA Panel et al., 2017). For botanicals in medicinal products, however, an alternative authorization is possible in the category of traditional herbal medicinal products (European Parliament and the Council of the European Union, 2004). For these products, safety and efficacy can be established by sources that indicate a long tradition of use of 30 years, of which 15 are within the EU (Committee on Herbal Medicinal Products, 2011; 2017). If such traditional use can be established, a botanical product can be authorized for placement on the market as a traditional herbal medicinal product (European Parliament and the Council of the European Union, 2004). As different stakeholders and member states started to question the seemingly arbitrary differential treatment under the food regulatory regime, it was deemed necessary that the Commission investigate this further. The evaluation of the botanical health claims was therefore put on hold, allowing for exploring the potential role of evidence on traditional use as substantiation for botanical health claims.

Because the assessment of botanical health claims is still on hold, these claims are subject to the transitional measures of the NHCR (European Parliament and the Council of the European Union, 2006). These transitional measures are usually temporary measures that provide FBOs with a timeframe to implement a new regulation and authorities to inplement procedures. Formerly, the use of trade names with reference to health also fell under such transitional measures (European Parliament and the Council of the European Union, 2006). Given that the procedures as laid down in the NHCR are not fully implemented, the transitional measures still apply for these approximately 2000 claims. This means these claims can be used in the communication of products under two conditions: (1) their application was submitted before 31 January 2008, and (2) the claim and its substantiation meet the general requirements of the NHCR and relevant national provisions of the EU member states. Based on Article 6 of the NHCR, these claims must thus be substantiated with generally accepted scientific evidence. It is, however, up to the member states what this evidence should entail and how it is evaluated (European Parliament and the Council of the European Union, 2006).

### 2.2 The role of case law in regulatory frameworks

Different consultations (EHPM and EBF, 2012; Anton et al., 2013), as well as the most recent regulatory fitness check (European Commission, 2019b; 2020), have not led to a solution for resuming the assessment of botanical health claims. Various CJEU cases were filed on health claims as such and addressed botanical health claims specifically. In the EU, cases from the CJEU can be used to further interpret legislation and ensure its consistent application throughout the European Union (The Member States, 2007). Any natural or legal person is allowed to start a proceeding against a regulatory act. A successful challenge must meet five conditions: (i) the body must be amenable to judicial review; (ii) the type of act in question must be open to challenge; (iii) the claimant must have a standing to act in that position (*locus standi*); (iv) the illegality must be in the scope of Article 263 TFEU; and (v) the time limits set in the treaty must be respected. An applicant is considered not to have *locus standi* if they are to no extent concerned with the request that is raised. A ruling of the CJEU is legally binding for the EU member states. It remains, however, up to national courts to apply and enforce the CJEU interpretations of EU law.

The rulings on botanical health claims have been used in national courts to interpret the legality of actions on the Dutch market. The CJEU rulings are furthermore used by self-regulatory institutions in the Netherlands, like the “Keuringsraad” or the Dutch Advertising Committee (DAC). These self-regulatory bodies must ensure a level playing field in advertising communications in the Dutch market. The advertising code on health products of the Keuringsraad has been reviewed and approved by the formal enforcement authority in the Netherlands, the Dutch Food and Consumer Product Safety Authority (NVWA) (NVWA & Keuringsraad, 2022). As such, self-regulatory institutes play an important role in regulating the health communications of food products in the Netherlands.

Although the status of botanical health claims has been unchanged since 2010, the CJEU case law and consequential national interpretation in national courts and self-regulatory actions have thus led to further clarification and interpretation of this specific group of claims falling under the transitional measures.

## 3 Methodology

The main aim of this study was to understand the role of CJEU case law on the implementation of the NHCR and, more specifically, the botanical health claims. It may furthermore highlight emerging issues from the implementation of this legislative act relevant to food products in general.

The impact of CJEU case law was analyzed in four steps. At first, case law of the CJEU was reviewed to understand the most pressing issues forwarded to the CJEU in relation to the NHCR, botanical health claims, and risk assessment, respectively. Second, national cases addressing (botanical) health claims and transitional measures were analyzed to clarify how national courts interpreted and applied the rulings from the CJEU and how this was connected to enforcement. Third, the decisions from DAC were subsequently reviewed to obtain a more practical interpretation and application of the CJEU case law.

Finally, academic literature on the topics emerging from the reviewed cases was reviewed to gain deeper insights into the implications of the NHCR and case law.

### 3.1 Case law selection

Judgments of the CJEU were retrieved via the Eur-lex and Curia databases using the following search terms: “health claims” and “botanicals.” Cases that concerned the risk assessment process or the practical use of specific health claims toward consumers were included. Cases concerning general health claims, claims used prior to the implementation of the NHCR, or cases that concerned information outside the scope of this review were excluded. One additional case was excluded as this concerned the annulment of recitals of Commission Regulation (EC) No 432/2012 and the register of health claims, which are not binding legislative acts. A list of excluded cases can be found in Supplementary Table 1.

All relevant cases were analyzed to understand the case subject, the formulated requests, the CJEU’s arguments, and the final ruling. The CJEU’s reasoning was used and extrapolated to the implications of that case for the interpretation and application of the NHCR. Relevant cases included requests for preliminary rulings, appeals from rulings of the General Court, claims on failure to act, and claims on actions for annulment. A total of 14 individual cases and one joint case were analyzed, including preliminary rulings, appeals, actions for failure to act, and actions for annulment.

The national case law was retrieved from the Dutch public court decision databases, accessed in June 2024. The cases were accessed using the following search strategies: “health claims AND botanicals,” “Health claims AND food supplement,” and “aandieningscriterium AND botanicals.” Only cases that took place after the publication of the permitted list in Commission Regulation No 432/2012 were included. There were no limitations as to the regulatory framework of the cases, which included food and medicinal law, commercial practice law, customs law, and tax law. Cases were included when they referred to or were ruling on similar issues as identified in the CJEU cases or when new emerging issues were found. Cases were excluded when they concerned specialized food or regulatory frameworks outside the scope of this review (e.g., military cases or business disputes). The included and excluded cases are displayed in Supplementary Table 2. Both search strategies resulted in the inclusion of 17 cases.

The decisions from the Dutch Advertising Committee were retrieved from their website, which includes a database with all past cases. The database was searched using the following search terms: “food supplement and health claim.” Similarly to the national case law, only decisions from after 2012 were included in the analysis. Like the Dutch national court cases, inclusion followed when the cases concerned CJEU or new emerging issues on the risk assessment of (botanical) health claims. Cases falling under other regulatory frameworks (e.g., veterinary products) or referring to health claims prior to the implementation of the NHCR were excluded. Supplementary Table 3 provides the in- and excluded DAC cases. The search resulted in 31 included decisions of the Dutch Advertising Committee.

Cases were excluded for inclusion after initial review when they were referring to substances, such as feed, cosmetics, or others.

### 3.2 Case law analysis

The analysis commenced with the review of the cases from the CJEU that provided (preliminary) rulings on health claims, risk assessment, and botanicals. The main issues were identified and used for the further analysis of the national and DAC cases.

National cases were included in the review when they either used CJEU case law in argumentation or when similar topics were addressed in the argumentation or decision. Following inclusion, cases were analyzed in-depth to understand the argumentation and regulatory framework applied to the dispute. To what extent the argumentation by the CJEU and the national courts were similar was assessed and, if different, what the reasoning was to deviate from existing case law. In line with national cases included, rulings from the self-regulatory authority, although not legally binding, also provide insight into the use of the regulatory framework and the case law for the self-regulation of foods. These cases were reviewed to gain further insight into the more practical consequences of these cases. Several cases were used in this review as illustrations to allow for an understanding of the line of argumentation followed by the Dutch national court and the DAC.

Finally, the topics and argumentation were reviewed with literature from, amongst others, legal scholars, nutritional sciences, and economic scholars. The literature was searched based on the five emerging issues identified from the case law analysis.

## 4 Results

**FIGURE 1:**
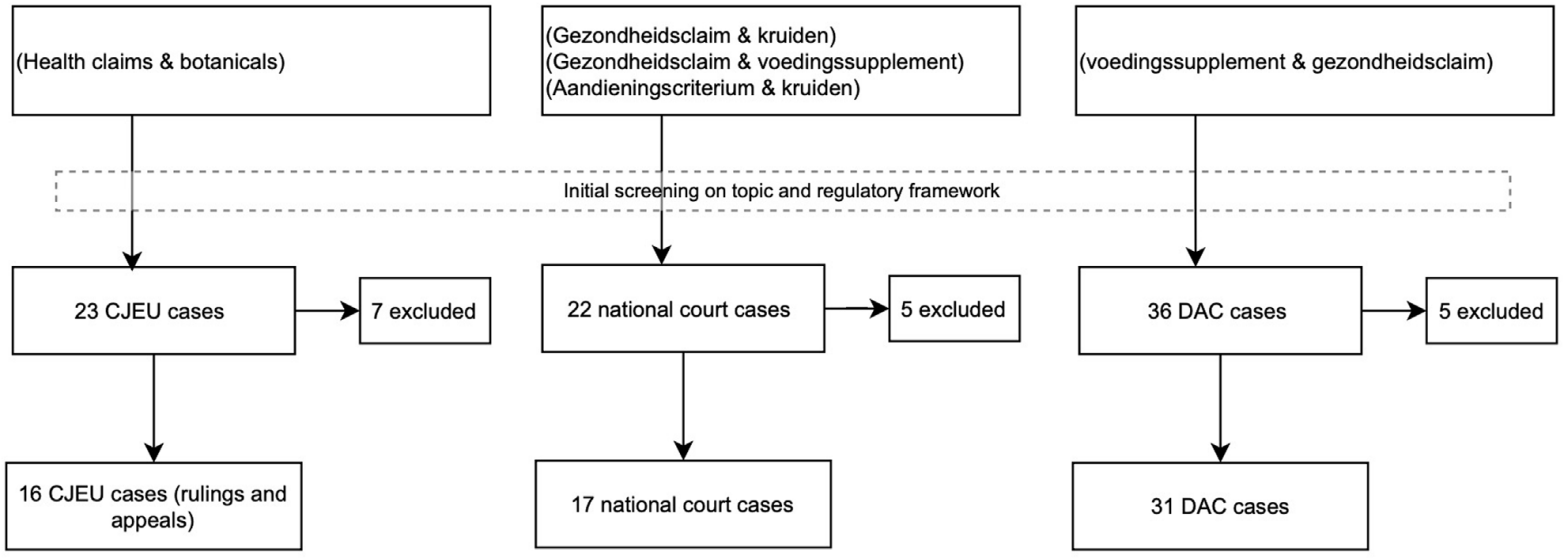
Cases included after the search and initial review and following the full case review.

An overview of the reviewed cases that contributed to the understanding of the emerging issues is illustrated in Figure 1.

The analysis of the CJEU cases led to four emerging issues: the classification of food and medicinal products, the definition of health claims, general evidence requirements, and evidence requirements for claims falling under the transitional measures. One emerging issue from the national and DAC cases was identified in commercial communication in the online environment.

### 4.1 Is it a food or a medicine?

CJEU *Case C-140/07 Hecht-Pharma GmbH v Staatliches Gewerbeaufsichtsamt Lüneburg* is mainly known for its impact on the classification of medicinal products and food products (Court of Justice of the European Union, 2009). This preliminary ruling, requested by the German federal administrative court, concerned the classification of red yeast rice, which is known to contain small amounts of monacolin K. Monacolin K is known for its effect on the reduction of cholesterol, which is a known pharmacological effect. In this case, the Court ruled medicinal products by function are those products eliciting effects designed to make a medical diagnosis or to restore, correct, or modify physiological functions. The Court also specifies that the dose matters, as not every substance that can elicit a physiological response should be considered a medicinal product by function. Products that are presented as having properties for the prevention or treatment of disease are known as medicinal products by presentation and also fall within the definition of medicinal product as specified in Directive 2001/83/EC Art. 1.2.a (Court of Justice of the European Union, 2009).

In *Case C-177/15 Nelsons GmbH v Ayonnax Nutripharm GmbH and bachblütentreff Ltd.*, the classification of products under the transitional measures was addressed (Court of Justice of the European Union, 2016a). In this case, a product was previously considered a medicinal product but was now, under the NHCR, considered a food product. This classification issue was a result of the interpretation of medicinal products in case C-140/07. In this case, the Court ruled that because it was now categorized as a food product, the NHCR applies to the product (Court of Justice of the European Union, 2016a).

Although the Hecht-Pharma case further defined the classification of foodstuffs and medicinal products, three national cases further explained the concept of medicinal products by function: two specifically on products containing melatonin and one on a product containing glucosamine and chondroitin.

In the Netherlands, the Healthcare Inspectorate (IGZ) and the NVWA issued a general classification for all products containing 0.3 mg melatonin in a single dose as medicinal products in 2011. The authorities consider it sufficiently substantiated in the literature that melatonin significantly influences physiological functions. In the two national cases, the Dutch industry association for food supplements questioned the validity of the Dutch government taking this general stance. The first case, no 2788332, was predominantly content related and addressed the legality of imposing this general measure on a product category (Rechtbank Gelderland, 2015). For this, the decision was reviewed on several criteria. The court ruled that it was not recent, the case was dated 2015, but authorities had regarded doses of above 0.3 mg melatonin as medicinal since 2011. Additionally, the authorities were judged to have sufficient evidence to defend their viewpoint and enforcement measures based on this view. Finally, the measure was also not considered to be too general: the authorities differentiated between products based on the dose. Not all products containing melatonin are considered to be medicinal products; only those containing 0.3 mg of melatonin or more (Rechtbank Gelderland, 2015).

In the second case, no C/09/489402/HA ZA 15-635, another industry association claimed that the Hecht-Pharma case required the appraisal of products on a case-by-case basis and that consequently, the general policy instated here for all products containing melatonin was against this arrest. The court saw differently, as this stance would not allow for any general policies on products (Rechtbank Den Haag, 2016).

A third national court case, no AWB-21_2502 and 21_5482, appealed a decision to classify glucosamine- and chondroitin-containing supplements as medicinal products (Rechtbank Zeeland-West-Brabant, 2022). The court ruled here that a product is only classified as a medicinal product by presentation if there is any doubt about the functionality of a product in an individual’s diet. In this case, the product was labeled as a food supplement, and a statement that the product should not be considered a substitute for varied nutrition was sufficient indication that the product is used to supplement the diet and should thus be classified as a food product. The claims that were considered medicinal were false and not allowed but did not lead to the conclusion that the product is a medicinal product (Rechtbank Zeeland-West-Brabant, 2022).

The use of medicinal claims, and thus the sales of medicinal products by presentation, was also observed in the DAC cases (Reclame Code Commissie, 2018; 2023b).

#### 4.1.1 Classification because of customs tariff

In the EU, businesses need a specific license to produce and sell medicinal products. As a consequence, the classification of a food supplement as a medicinal product can become economically negative for an FBO because they can either not sell the product if they do not have a license or need to invest in obtaining the license. National case law, however, unveiled that importing products classified as medicinal products is preferred over importing them as food supplements. Medicinal products are also defined by the Customs Administration as products having a therapeutic or prophylactic effect. Different import rules and customs tariffs are enforced for these products.

Two national cases on the import of products with vitamins illustrate this different classification, where one product was classified as a food supplement and the other product as a medicinal product, although the bioactive ingredients were partially similar. These products were initially considered to be food supplements. It was up to the business to prove that the effects of these products were considered therapeutic or prophylactic. This must be substantiated by literature or otherwise.

The first case, no AWB-21_6600, was about supplements with vitamins D and K, which were presented as prevention products against deficiencies (Rechtbank Noord-Holland, 2024c). According to the FBO, a deficiency in vitamin D can lead to osteoporosis or sarcopenia in older people or rickets disease in children. The court decided that merely the prevention of vitamin deficiency would not classify a product as a medicinal product, as vitamin status says something about an individual’s general health. The therapeutic or prophylactic effect must be indicated in the label, literature, or otherwise. For this specific product, there was no such information on the label. Literature and otherwise did not provide evidence to support the necessity of the consumption of this product in the treatment or prevention of disease (Rechtbank Noord-Holland, 2024c).

In the second case, no AWB-21_6599, the FBO was able to provide sufficient evidence for the claim that vitamin C had to be regarded as a medicinal product (Rechtbank Noord-Holland, 2024b). As in the previous case, the prevention of vitamin deficiency was also not considered to be sufficiently valid to classify it as a medicinal product. A sufficient vitamin level was considered a general health status and not necessarily a disease outcome. The decisive information that led to the classification of the product as a medicinal product was the role of vitamin C in the prevention of scurvy and Barlow disease (Rechtbank Noord-Holland, 2024b).

One national court case, no AWB-21_6598, was an appeal on the import of ubiquinol (Rechtbank Noord-Holland, 2024a). The appellant indicated the product is a medicinal product that can be used to prevent ubiquinol deficiency and in the prevention and treatment of cardiovascular disease, chronic inflammation, diabetes type II, and neurodegenerative diseases. The product packaging, however, stated the product is a food supplement. The packaging did not provide any information on the therapeutic or prophylactic effects. A medicinal product by presentation must have the therapeutic or prophylactic effects visible on the “label, literature or otherwise.” As this was not the case for this product on the label and the appellant was not able to provide evidence in “literature or otherwise,” the appeal was dismissed, and the product was classified as a food supplement (Rechtbank Noord-Holland, 2024a).

It is important to note here that the court considered several aspects: the trade license obtained for the sales, distribution, or advertising of a medicinal product, the pharmacy-only status of the product in some jurisdictions, or the product classification already seen on the market. The inclusion of a leaflet or any other form of communication alongside a multivitamin product where reference was made to disease was also mentioned specifically. The court regarded these, however, to be indicative and not decisive. DAC cases were not found in this specific topic among the commercial cases evaluated.

### 4.2 Defining health claims and their scope

Three CJEU cases contributed to the further interpretation and definition of health claims within the context of the NHCR. The most well known and often-cited case is *Case C-544/10 Deutsches Weintor eG v Land Rheinland-Pfalz*, in which the statement “gentle acidity/easily digestible” was reviewed to determine whether it would fall under the definition of a health claim under Art. 2 (2) (5) of the NHCR (Court of Justice of the European Union, 2012). The FBO argued that the statement refers to general well-being and not health, whereas the national court considered that the definition of health claims should be broadly interpreted, leading to the used statement being considered a claim. The CJEU confirmed a broad interpretation of the definition of health claims, including references to products or substances as “less harmful” and both long-term and short-term health effects. The stated positive effect could persuade a consumer to purchase a product and should thus be considered as a statement falling within the scope of the NHCR (Court of Justice of the European Union, 2012). In *Case C-299/12 Green Swan Pharmaceuticals CR, a.s. v Státní zemědělská a potravinářská inspekce, ústřední inspektorát,* the CJEU was asked to determine whether claims fall under the definition of a reduction of disease risk claim when the reduction of a risk factor of the disease is not explicitly mentioned (Court of Justice of the European Union, 2013). The CJEU reasoned that the suggestion or implication of an effect on a risk factor makes it a health claim, irrespective of whether it is indicated how significant the reduction may be (Court of Justice of the European Union, 2013). In *Case T-17/12 Hagenmeyer & Hahn v European Commission*, the decision to reject the authorization of a disease risk reduction claim related to drinking water and the reduction to the risk of dehydration was appealed (Court of Justice of the European Union, 2014). The proposed claim was rejected as it was not considered to be sufficiently linked to the reduction of an actual risk factor for disease development. The CJEU stated that the risk factor component of the definition in Art. 14 (1) (a) cannot be ignored. Any claim authorized under this Article requires a designated risk factor in the development of disease, which also allows for distinguishing such disease risk reduction claims from medical claims on the treatment, prevention, and cure of disease (Court of Justice of the European Union, 2014).

The definition of health claims and the scope in which the NHCR applies has been the subject of two national court cases and five DAC cases. The argumentation in these cases often follows the conclusions of the Weintor case and the subsequent broad interpretation of the definition of health claims (Reclame Code Commissie, 2016a; 2016b, 2020c).

Two national court cases, no ROT 22/4631 and no 19/463, addressed the definition of health claims related to glucosamine-chondroitin products and the effect they have on joint health (College van Beroep voor het Bedrijfsleven, 2021; Rechtbank Rotterdam, 2023b). In both cases, not necessarily the products but the substances in the products were used in information on the maintenance of joint health. The court here argues that although no direct claim was made, the NHCR also covers the implication or suggestion of a connection between a food constituent and health. Given the Weintor case and the judgment that the definition of a health claim should be broadly interpreted, the indirect implication was considered a health claim in this case (College van Beroep voor het Bedrijfsleven, 2021; Rechtbank Rotterdam, 2023b).

The definition of health claims mainly focused on the distinction between general information on health and health claims in the DAC cases. In one DAC case on joint health, a complaint was raised about the product name, “joint support” (Reclame Code Commissie, 2021). The product name was believed to be a health claim, but it was not sufficiently clear how this product would lead to a positive health effect. In this specific case, the DAC concluded that although there are approved claims for vitamin C, vitamin D, and zinc in relation to joint health, which are also in the product, the product name was not sufficiently related to these nutrients. This could be confusing for consumers and thus mislead them. The FBO was consequently requested not to advertise the product in this way anymore (Reclame Code Commissie, 2021).

### 4.3 Evidence requirements for health claims

In *Case T-296/12 Health Food Manufacturers’ Association and Others v Commission*, the annulment of Commission Regulation (EU) 432/2012 and the list of on-hold claims was requested as it was deemed to be based on “improper assessment criteria” (Court of Justice of the European Union, 2015a). The CJEU ruled that there was sufficient legal ground, specifically the term “relationship” in article 2.5.5, to allow for the use of assessment criteria by the risk assessor. The specific requirements regarding the identification of the particular food that causes the effect are deemed necessary to fully understand the scientific substantiation and assess its relevance (Court of Justice of the European Union, 2015a).

*Case T-334/12 Plantavis GmbH and NEM v Commission* described several requests, of which one was on the annulment of the opinions published by EFSA (Court of Justice of the European Union, 2015b). However, the Court stipulated that the opinions of EFSA merely represent “intermediary” steps in the procedure and do not have any legal effects. Thus, they cannot be part of an action for annulment within the scope of Article 263 TFEU (Court of Justice of the European Union, 2015b).

One specific unauthorized health claim was subject in *Case C-296/16 Dextro Energy GmbH&Co.KG v Commission,* which appealed *Case T-100/15 Dextro Energy v Commission* (Court of Justice of the European Union, 2016c; Court of Justice of the European Union, 2017a). In the case, the annulment of Commission Regulation 2015/8 on the refusal to authorize five health claims, including a claim on glucose and the positive effects on the energy-yielding metabolism, was requested. This specific claim was positively assessed by EFSA but not authorized by the Commission on the grounds of general nutrition and health considerations. It was believed that such claims would pose a conflicting message with the general recommendation to reduce the consumption of sugar. The court ruled that messages that are potentially misleading cannot be protected under the freedom of speech. The principles of proportionality were not infringed as the NHCR specifically allows the Commission to weigh aspects of political, economic, and social perspectives in their decision-making. This case thus confirmed that the opinion issued by EFSA is merely one element that is considered by the Commission and not a legal act that can be disputed in court (Court of Justice of the European Union, 2016c; Court of Justice of the European Union, 2017a). The practical interpretation of the evidence requirements was addressed in several DAC cases in which particular studies were reviewed in line with the assessment criteria and CJEU rulings (Reclame Code Commissie, 2012). These cases followed the EFSA criteria, and no additional evidence types were deemed sufficient to substantiate health claims.

### 4.4 Claims falling in the transitional measures

In *Case C-363/19 Konsumentombudsmannen v Mezina AB*, a preliminary ruling was requested to determine whether specific function claims and related general health claims should be substantiated with scientific evidence and, were that to be the case, where the burden of proof would lie (Court of Justice of the European Union, 2020). The CJEU here ruled that claims falling under the transitional measures, including botanical claims, must fulfill the applicable general requirements laid down in the NHCR and national provisions. It is up to the users of the claim to justify them, meaning the burden of proof lies with the FBOs. The Court furthermore stipulates that the NHCR does not provide any clarification as to what the scientific evidence, which is consequently dealt with in national law, should entail (Court of Justice of the European Union, 2020).

Other court actions have also touched upon the transitional measures. In *Case T-296/12 Health Food Manufacturers’ Association and Others v Commission*, the applicant requested the annulment of Commission Regulation (EU) No 432/2012 and Commission Decision of 16 May 2012 that resulted in a list of permitted claims and a list of on-hold claims (Court of Justice of the European Union, 2015a). The applicants argued that the Commission failed to achieve the compiling of one list because of the on-hold claims on botanicals. However, the court ruled that a list being incomplete and composed gradually was not against the requirements laid down in Article 13 of the NHCR. It did not lead to any legal uncertainty, and thus, no action was required on either the positive list or the on-hold botanical claims. Another request for the annulment of Commission Regulation (EU) 432/2012 was made in Case T-334/12. The applicants were, however, not concerned by Commission Regulation (EU) 432/2012, and the request was consequently denied (Court of Justice of the European Union, 2015a).

Lastly, two appeal cases also referred to the transitional measures for health claims. *Case C-637/15P VSM Geneesmiddelen BV v Commission* appealed General Court decision *Case T-578/14* (Court of Justice of the European Union, 2015c)*,* in which VSM claimed that the Commission failed to act as it had not asked EFSA to continue the assessment of the botanical health claims (Court of Justice of the European Union, 2016b). The Court ruled, however, that the “act” in this sense referred to taking a position or defining a position as per Article 265 TFEU. Although the Commission did not satisfy the wishes of the applicant, it did take a decision that led to a sufficiently equal condition and no legal uncertainty. In the appeal in Case C-637/15 P, the appellant requested to set aside the previous ruling and used the same claims. The Court concluded all grounds of appeal were inadmissible: on-hold claims do not lead to legal uncertainty, and the current situation may be more advantageous than a situation in which the claims are assessed (Court of Justice of the European Union, 2016b). The appeal in Joined *Cases C-596/15 P and C-597/15 P* (Court of Justice of the European Union, 2017b) appealed the General Court decision in *Cases T-619/14 and T-620/14,* which the appellants also viewed as an infringement of the Commission because it had not requested EFSA to resume the assessment (Court of Justice of the European Union, 2015d; 2015e). The actions were deemed inadmissible because the applicants were not sufficiently concerned with transitional measures. They were not producers or sellers of botanical products and thus lacked locus standing. Furthermore, they could not argue how the adoption of a positive list, and thus resuming the evaluation, would benefit them. All grounds for appeal were thus deemed inadmissible (Court of Justice of the European Union, 2015d; 2015e).

No national cases addressed the botanical health claims under the transitional measures, but the evidence requirements for on-hold claims have been discussed in multiple DAC cases. The decisions in these cases follow the conclusions that health claims on the on-hold list can be used but also unveil the national provisions that can apply. All DAC cases follow a similar line of reasoning. FBOs must have scientific evidence available that justifies the claimed effect. If this cannot be sufficiently achieved, claims can only be made under certain conditions. In practice, this overall condition is translated into the use of a disclaimer for such products (Keuringsraad, 2019). By using the disclaimer, for example, “evaluation health claim is pending” or “health claim is awaiting European approval,” FBOs put a reserve on their claim and inform consumers about the on-hold status of the claim, thus avoiding deception. FBOs must still be able to provide scientific evidence upon request.

In one of the cases, 2015/00916 – CVB, the board of appeal refers to a published scientific opinion (Reclame Code Commissie, 2015). In this case, a claim is made about green tea and the stimulation of fat metabolism. In the initial case, the DAC concluded that the FBO did not provide sufficient evidence to substantiate the claim. In the appeal, this conclusion was maintained, and the board referred to the scientific opinion from EFSA on green tea, which had previously indicated that there was no cause-and-effect relationship established between green tea and weight management or fat metabolism. The board, therefore, concluded that this claim may be on hold but can “apparently not be proven” (Reclame Code Commissie, 2015). The use of this published scientific opinion to not allow for the use of a specific claim illustrates how national provision can differ from EU policies. Even though the claim has been assessed, it still falls under the transitional measures and is thus allowed to be used following EU standards. The Board of Appeal, however, concludes that the claim cannot be used based on this assessment.

### 4.5 Commercial communication in the online environment

An emerging issue from the included national and self-regulatory case law was the provision of information in the online environment (Reclame Code Commissie, 2020a; Rechtbank Amsterdam, 2022). In the Dutch court case, a drug store was given a fine for breaching medicinal law by presenting and selling a product as a medicinal product whilst not having a license for it (Rechtbank Rotterdam, 2023a). Another case concerned the reviews of a product (Rechtbank Amsterdam, 2022). In those reviews, reference was made to medicinal effects, whilst other information did not make sufficiently clear that the product is a food supplement (Rechtbank Amsterdam, 2022). The applicant in this case, who was initially fined for the information in the reviews, argued that she could not be held responsible for reviews that were not written by her. The Court reasoned differently and stated that an FBO is responsible for ensuring the information on a website is in line with applicable rules and regulations. Reviews are considered product information, and it is an FBO’s responsibility to moderate the reviews to ensure compliance with existing legislation (Rechtbank Amsterdam, 2022).

Several DAC cases concerned the use of unauthorized health claims and medicinal claims in testimonials that were accessible on the website (Reclame Code Commissie, 2023a) or in the product reviews on a website (Reclame Code Commissie, 2020a). Unauthorized statements and third-party webpages, such as those of independent distributors (Reclame Code Commissie, 2016a) or a forum linked to a product’s page (Reclame Code Commissie, 2013b), have also been brought to the DAC. Similarly, printed information on a food supplement, including statements on its benefits, provided alongside a medicinal product has been the topic of discussion in a case brought to the DAC (Reclame Code Commissie, 2013a). In all cases, the DAC ruled that the information on the leaflet was considered a claim, either an unauthorized health claim or a medicinal claim. This is in line with the broad interpretation of health claims from the CJEU cases and the CJEU case on the communication of health claims by healthcare providers (Reclame Code Commissie, 2013a).

Two DAC cases requested a decision on the provision of information via an advertorial (Reclame Code Commissie, 2020b; 2023c). The *Oxford Learner’s Dictionary* defines an advertorial as “an advertisement that is designed to look like an article in the newspaper or magazine in which it appears” (Oxford Learner’s Dictionaries, 2024). For one of the advertorials, the complaint was about general false and misleading information. The article described the difference between synthetic and natural vitamin C and the general necessity to consume vitamin C (Reclame Code Commissie, 2023c). In the other case, a product containing vitamin A was promoted as a product for maintaining healthy mucous membranes (Reclame Code Commissie, 2020b). Although the information was provided in the form of an article, it was still sufficiently linked to a specific product for it to be regarded as a health claim. The DAC believed both advertorials to be subject to the NHCR and the subsequent advertising guidelines issued by the DAC. In the case of vitamin C, the DAC ruled that the information in the advertorial was against both specific and general premises of the NHCR (Reclame Code Commissie, 2023c). The advertorial, consequently, did not follow the advertising code. In the second case on vitamin A and healthy mucous membranes, the claim was allowed, although the wording was not in line with the allowed translations of the authorized health claim (Reclame Code Commissie, 2020b). Other claims in the advertorial were based on the on-hold claims that were not sufficiently substantiated by the FBO, and no disclaimer was provided alongside the claims (Reclame Code Commissie, 2020b). These claims were thus deemed unallowed and cannot be used again by the FBO.

## 5 Discussion

The main aim of this study is to understand how CJEU case law on health claims and botanicals shaped the interpretation and application of the NHCR and, more specifically, the transitional measures. As shown in the results section above, rulings by the CJEU, national courts, and self-regulatory bodies in the Netherlands have provided a clarification on the interpretation of the classification of food and medicinal products, the definition of health claims, the evidence requirements for health claims in general and more specifically for claims falling under the transitional measures and the commercial communication in the online environment. The effect of CJEU rulings is highlighted in the usage of their conclusions in the argumentation of the more practical national cases and self-regulatory DAC cases. These rulings have thus contributed to both a clarification of the regulatory framework and shaped the food information environment for voluntary food information.

### 5.1 Foods or pharmaceuticals in products and claims

As shown by the rulings in Cases C-140/07 and C-544/10, case law has further established the classification of food and medicinal products as well as health claims and medicinal claims. The classification of a product is based on the effect a product (by function for a medicinal product) has or the effects it presents to have (by presentation for a medicinal product) (Nicoletti, 2012). The Dutch national court case further clarified that presenting the medicinal effects of a product, even though its physiological effect is known to not be related to curing, treating, or preventing disease, would classify a product as a food product (Rechtbank Gelderland, 2024). Hence, not every presented medicinal effect immediately leads to a classification of a product as a medicinal product; it can also merely be an illegal claim. In other cases, in which the wording referred to the treatment, prevention, or cure of a disease and thus referred to the medicinal or pharmacological effects of a product, the court or DAC ruled that the product would classify as a medicinal product by presentation.

Although the court cases provided additional insights into the argumentation to classify a product as a food or medicinal product, classification still remains somewhat of a gray area: it remains the responsibility of individual member states. In their decisions, other aspects, such as culture, can be weighed in this classification, which may lead to differences among member states in product status (Silano et al., 2011). This also became apparent in the Dutch cases on melatonin. Following Commission regulation No 432/2012 on the authorized health claims, a 0.5 mg dose of melatonin would be considered a food that can support in relieving subjective feeling of jetlag, and for a dose of 1 mg of melatonin, a claim can be made to reduce the amount of time it takes to fall asleep (European Parliament and the Council of the European Union, 2012). These are authorized health claims based on doses over 0.3 mg, which may be considered foods in certain countries, but products containing this amount of melatonin would be regarded as medicinal products in the Netherlands. The different court rulings indicated that the Dutch authorities have rightfully established the maximum amount of melatonin in foodstuffs (Rechtbank Gelderland, 2015). Products with higher amounts of melatonin are thus medicinal products by function in the Netherlands, even though they may be classified as foodstuffs in other member states. Clarification of the definition of food and medicinal products by CJEU rulings has thus not fully resolved these classification differences.

The classification of food products and medicinal products and their claims have been criticized before. Whereas these two product categories seem to be strictly separated, the evidence requirements, including the research methodology to show the beneficial effects, are highly similar (de Boer et al., 2014; Todt and Luján, 2017; Lenssen et al., 2022). Food products, including botanicals, can contain multiple bioactive substances, of which synergistic effects cause beneficial effects, or one substance that has multiple but subtle positive effects on health (Heaney, 2006; Bast et al., 2013). Short-term intervention trials would not show these effects, although long-term consumption may be beneficial.

The classification of products, although further clarified in case law, will remain subject to discussion because of differences among member states and the complexity in differentiating between pharmacological and physiological effects. The analysis of case law did show that communicating effects a product cannot elicit, such as claims on preventing, treating, or curing disease, results in the statements being considered illegal health claims or making the product a medicinal product by presentation.

### 5.2 Scientific evidence is mandatory to substantiate claims, including those falling under the transitional measures

In CJEU case C-363/19, the evidence requirements for claims falling under the transitional measures were further clarified: on-hold claims must be substantiated with scientific evidence. That general requirement applies to all claims that fall within the definition of a health claim. The assessment of the scientific evidence by EFSA is not a legal act on which legal actions via the CJEU are possible. The assessment is considered an intermediary step in the process in which the final authorization decision published in a commission regulation is the formal legislative act. The assessment criteria used by EFSA are appropriate, given that the scientific evidence must establish a relationship between food and health.

The NHCR requires, per Art. 6, that health claims are substantiated with generally accepted scientific evidence (European Parliament and the Council of the European Union, 2006). In one of the CJEU cases on the transitional measures, this was further interpreted as evidence that cannot be limited to “beliefs, hearsay derived from popular wisdom, or the observations or experiences of persons outside the scientific community” (Court of Justice of the European Union, 2020). Although this ruling does further clarify the requirements for claims falling under the transitional measures, it still does not provide any insights into the way forward regarding the risk assessment of the claims that are currently on hold. Especially with the individual member states being responsible for the assessment of the scientific substantiation of these claims falling within the transitional measures (Court of Justice of the European Union, 2020), member states may approach this differently. This is exemplified by the Dutch authorities introducing a disclaimer that is currently not implemented in other member states (Keuringsraad, 2019). It could be argued that, based on these CJEU conclusions, evidence of traditional use alone cannot be considered generally accepted scientific evidence. However, it does also not fully dismiss its use. Hence, traditional use evidence could be derived from scientific disciplines other than nutritional sciences, such as history. The risk assessment in its current form, however, requires the establishment of a cause-and-effect relationship (de Boer et al., 2014; Lenssen et al., 2018). Such a strong scientific base for a statement cannot be derived from historical research but would require human intervention trials (Lenssen et al., 2020). Given this requirement, evidence based solely on traditional use would not be sufficient substantiation of botanical health claims.

A tiered evidence approach has been suggested by the European industry association for health products in 2021 (European Federation of Associations of Health Product Manufacturers, 2021). In this approach, it is suggested that different tiers of evidence lead to different types of assessment, subsequentially leading to authorization of differently phrased claims. One of these tiers could be authorization based on traditional use evidence using the wording “x is traditionally used for y.” This would require a different approach for both risk assessment and risk management. Currently, EFSA uses a relatively clear approach, and health claims with a negative opinion from EFSA are not authorized by the Commission. In order to implement a graded evidence approach, the evaluation and the authorization would need to be adjusted: new assessment criteria are required for studies other than those from nutritional sciences, and risk management would need to establish how these evaluations are to be used in the authorization process.

The botanical claims have already been on hold for more than 10 years, and the needed political decision on how to move forward is eagerly awaited in the field. Although some direction is provided by case law, a more thorough decision is required to fully resume the evaluation of botanical health claims.

### 5.3 Sufficient legal certainty yet regulatory uncertainty for botanical health claims

Several CJEU cases have addressed requests for the Commission to ask EFSA to continue the evaluation and rulings on the Commission’s failure to act on the on-hold status of the botanical health claims. There is, however, no legal ground upon which the CJEU could force action from the Commission to request EFSA to finalize the assessment of the botanical health claims. Therefore, these cases did not change the status of the botanical health claims.

The Commission did a regulatory fitness check of, amongst others, the General Food Law and the NHCR (European Commission, 2019a; 2020). One of the aims of this review was to understand whether the regulations are meeting their objectives. For the review of the NHCR, the botanical health claim was one of two main subjects. The fitness check concluded that as long as all botanical health claims fall under the transitional measures, the objectives are not fully met. Differences in the substantiation requirements among member states may negatively impact the internal market. Additionally, there may be claims on the market that cannot be substantiated with the required level of evidence. Hence, consumers may be exposed to misleading claims. This was also concluded by AG Bobek, who considers claims from the permanent regime different from those under the transitional regime (Bobek, 2017). The NHCR’s objectives, ensuring the optimal functioning of the internal market and creating the highest level of consumer protection, may thus not be met. Although the CJEU repeatedly concluded that there is sufficient legal certainty created by the transitional measures—as business operators know that these transitional measures exist and the measures as such are clear—there is uncertainty on the effectiveness of the regulation. Not knowing when EFSA will resume its assessment of the health claims on hold and under what conditions leads to uncertainty, which may negatively impact innovation in the long run (Lenssen et al., 2018).

For botanicals in food specifically, another aspect of interest in different member states is the safety of these supplements. Although the NHCR regulates the communication of food product benefits, some botanicals have known side effects or risks (Greeson et al., 2001; de Heer, 2024). For medicinal products, information on potential adverse events must be provided in a leaflet, and a pharmacovigilance system must be in place to monitor the side effects of medicinal products (European Parliament and the Council of the European Union, 2001). This has led to an overall ban on certain botanicals, such as kava. The Dutch authority for public health and the environment recently looked into several botanicals to understand their benefits and risks (Chen, 2024; de Heer, 2024; de Heer and de Wit-Bos, 2024; de Heer et al., 2024). They concluded that some botanicals, like ashwagandha, may pose risks to consumers. To optimize not only the communication of health benefits but also improve the communication of risks, the Dutch authority has suggested a vigilance system for botanicals to gather information, monitor side effects, and ensure the provision of information (Bureau Risicobeoordeling & onderzoek, 2024). In Belgium, France, and Italy, the BELFRIT program aimed to create positive or negative lists of botanicals that can or cannot be part of food products (Cousyn et al., 2013). These lists were also mainly based on the safety of these products.

The current status of botanical health claims currently suffices for FBOs in terms of legal certainty. At the same time, the regulatory fitness check concluded that the objectives of the NHCR may not be met: it remains uncertain how the field will be impacted by measures that are still expected to be taken. It is particularly questioned to what extent companies are affected in their innovation plans due to the uncertainty on how this regime will be continued (European Commission, 2020). This again emphasizes the clear need for a decision to be made on the substantiation of botanical health claims. This decision should follow both the objectives of the NHCR and the interpretation of the CJEU of the NHCR. Simultaneously, the protection of consumers should also be viewed in light of the risks of botanicals, raising the possibility for dedicated legislation for this product category dealing with safety and efficacy.

## 6 Conclusion

The CJEU rulings, national court cases, and self-regulatory DAC output have clarified how the NHCR needs to be interpreted and implemented. They have shed light on multiple issues, including what statements we understand as being a health claim and how the transitional measures for botanical claims need to be seen from a legal perspective, including the necessity of scientific evidence to substantiate claims falling under these measures. The court rulings, together with the Regulatory Fitness and Performance (REFIT) evaluation of the NHCR, have particularly highlighted that having a substantial group of on-hold claims negatively affects the market and impacts innovation. Although there is no legal uncertainty as such, the internal market may currently not function optimally as two different types of claims are found on the market: those that are scientifically sound and those that have not been assessed on their scientific merit. Consumers may, therefore, be exposed to claims for which no objective scientific evidence is available. The CJEU confirms that the assessment criteria for scientific evidence used by EFSA are appropriate and even indicates that merely non-scientific evidence, like experiences or hearsay, cannot sufficiently substantiate botanical health claims. However, this does not fully close the door to other types of scientific evidence, such as historical or anthropological studies on the health benefits of botanicals.

The analysis of case law on botanical health claims highlights that case law does lead to further interpretation of the NHCR. Its effects are limited to legal interpretation, whereas a broader policy perspective is required to resume the assessment of the botanical health claims. Only a formal decision by the risk manager can resolve the impasse with these claims and lift the uncertainty faced by producers of products using these claims.

## Data Availability

The raw data supporting the conclusions of this article will be made available by the authors, without undue reservation.
