# Supplementary material for: Bridging regulation and practice: CJEU and Dutch case law on botanical health claims
Source: Front Pharmacol. 2025 Feb 19;16:1523904. doi: 10.3389/fphar.2025.1523904 (PMC11879797; doi:10.3389/fphar.2025.1523904)
Supplement: Supplementary file 1 [file DataSheet1.pdf]

Supplementary table 1. Excluded CJEU cases

This table contains the CJEU cases that were retrieved in the used search strategy but excluded from the analysis after review in chronological order. The table includes the case number, reason for exclusion from the analysis and the involved parties.

**Case number**	**Reason for exclusion**	**Involved parties**
	Reference for a preliminary ruling from the Bundesgerichthof (Germany) lodged on 4 February 2011.<br><br>Withdrawn	Schutzverband der Spirituosen-Industrie eV v Sonnthurn Vertriebs GmbH (Case C-51/11)
ECLI:EU:T: 2014:798	Annulment of several recitals of Commission Regulation (EU) No 432/2012 and the union register of health claims	Case T-354/12 Afepadi and Others v Commission
ECLI:EU:C: 2014:252	Application of NHCR on a slogan on dairy	Case C-609/12 Ehrmann AG v Zentrale zur Bekämpfung unlauteren Wettbewerbs eV
ECLI:EU:C: 2014:2335	Definition of organic products and labelling of organic products	Case C-137/13 Herbaria Kräuterparadies GmbH v Freistaat Bayern
ECLI:EU:C: 2015:823	Calculation of substance towards nutrition and health claims related to salt	Case C-157/14 Société Neptune Distribution v Ministre de l'Economie et des Finances
ECLI:EU:C: 2016:563	Application of the NHCR on the communication of information towards health care professionals	Case C-19/15 Verband Sozialer Wettbewerb eV v Innova Vital GmbH
ECLI:EU:C: 2020:60	Definition of general health claims regulated in art. 10.3 of the NHCR.	Case C-524/18 Dr Willmar Schwabe GmbH & Co.KG v Queisser Pharma GmbH & Co. KG

Supplementary table 2. Dutch national court cases

This table lists all national court cases retrieved from the search strategy in chronological order. A brief and general description of the case is given. When meeting the inclusion criteria, the involved parties are described and overall issue as identified in the analysis is provided.

**Case number**	**Case description**	**Involved parties**	**Overall issue**
ECLI:NL:CBB: 2012:BY050	Appeal<br><br>Use of medicinal claims and thus sales of medicinal products by presentation	Research centre vs Dutch minister of public health, well-being and sports	Classification of products
ECLI:NL:RBZWB: 2013:7350	Advertising for prescription only medicinal product	Excluded	Excluded
ECLI:NL:RBROT: 2014:2601	Use of unauthorised and/or misleading health claims	Retailer food supplements vs. producer OTC medicinal products and health products	Defining health claims
ECLI:NL:RBROT: 2014:1615	Use of medicinal claims and thus sales of medicinal products by presentation	Web shop with botancials vs Dutch minister of public health, well-being and sports	Classification of products
ECLI:NL:PHR: 2015:2466	Military case	Excluded	Excluded
ECLI:NL:RBGEL: 2015:1182	Classification of products containing over 0.3 mg melatonin as medicinal products	Appeal<br>Industry association vs. inspection for health care	Classification of products
ECLI:NL:RBDHA: 2016:9458	Cancellation of trade agreement between business	Excluded	Excluded
ECLI:NL:RBDHA: 2016:13311	Unlawful comparison	Excluded	Excluded
ECLI:NL:RBAMS: 2016:397	Advertising for medicinal products	Excluded	Excluded
ECLI:NL:RBDHA: 2016:6019	Classification of products containing over 0.3 mg melatonin as medicinal products	Industry association vs. Dutch government	Classifications of products
ECLI:NL:PHR: 2018:625	Classification of products as food supplements	Sex shop exploiter vs. state secretary of finance	Classification of products
ECLI:NL:RBAMS: 2019:3386	Use of statements that should be considered health claims which are not authorised	FBO vs. FBO	Defining health claims
ECLI:NL:CBB: 2021:180	Appeal<br><br>Provision of information that implies a health benefit	Retailer food supplements vs Dutch minister of public health, well-being and sports	Defining health claims
ECLI:NL:RBAMS: 2022:7416	Use of medicinal claims and thus sales of medicinal products by presentation	Health retailer vs. Dutch minister of public health, well-being and sports	Classification of products
ECLI:NL:RBZWB: 2022:6136	Use of medicinal claims and thus sales of medicinal products by presentation	Web shop of health products vs state secretary of public health, well-being and sports	Classification of products
ECLI:NL:RBMNE: 2022:632	Use of medicinal claims and thus sales of medicinal products by presentation	Retailer vs. Dutch minister of public health, well-being and sports.	Classification of products
ECLI:NL:RBROT: 2023:854	Use of claim that is not in the list of authorised health claims.	Seller of herbs, spices, tea, OTC medicinal products, nutrition, cosmetics and more vs. Dutch minister of	Defining health claims

		public health, well-being and sports.	
ECLI:NL:RBROT:2023:75	Use of medicinal claims and thus sales of medicinal products by presentation	Web shop with health products vs. NVWA	Classification of products
ECLI:NL:RBNHO:2024:3618	Establishing the customs tariffs by classifying the product is food supplement or medicinal product	Importer and seller of products for human consumption vs. customs	Classification of products
ECLI:NL:RBNHO:2024:3615	Establishing the customs tariffs by classifying the product is food supplement or medicinal product	Importer and seller of products for human consumption vs. customs	Classification of products
ECLI:NL:RBNHO:2024:3619	Establishing the customs tariffs by classifying the product is food supplement or medicinal product	Importer and seller of products for human consumption vs. customs	Classification of products
ECLI:NL:RBGEL:2024:4667	Use of medicinal claims and thus sales of medicinal products by presentatation	Retailer of food supplements vs. Minister of public health, well-being and sports	Classification of products

Supplementary table 3. Dutch advertising committee cases

This table lists all Dutch advertising committee cases retrieved from the search strategy in chronological order. A brief and general description of the case is given. When meeting the inclusion criteria, the overall issue as identified in the analysis is provided. Note: involved parties are not always disclosed and thus not provided in this table.

Case number	Case description	Overall issue
2012/00073	Advertisement of food supplements for which no product specific research is available but is based on research into individual product constituents.	Evidence requirements
2013/00135	Statement in an advertisement that could be an implication of a health benefit and would thus be subject to NHCR	Defining health claims
2013/00227	Use of medicinal claims and thus sales of medicinal products by presentation. No formal fines just breach of compliance of existing advertising code.	Classification of products
2013/00309	The provision of general information on health benefits of probiotics in combination with a trial product. This combination is considered to be an implicit claim and the information subject to the NHCR.	Commercial communication
2013/00311	Statement in an advertisement that could be an implication of a health benefit and would thus be subject to NHCR	Defining health claims
2013/00404	Use of medicinal claims and thus sales of medicinal products by presentation. No formal fines just breach of compliance of existing advertising code.	Classification of products
2013/00695	Use of medicinal claims and thus sales of medicinal products by presentation. No formal fines just breach of compliance of existing advertising code.<br><br>The FBO was asked to provide evidence to substantiate health claims made on the product. The evidence was reviewed by the Keuringsraad and deemed insufficient.	Classification of products<br><br>Evidence requirements
2013/00735	Claims are used on a substance for which there is no authorised health claim.<br><br>Users of a food supplement make statement on a website that are subject to the NHCR.	Defining health claims<br><br>Commercial communication
2014/00233	Use of medicinal claims and thus sales of medicinal products by presentation. No formal fines just breach of compliance of existing advertising code.	Classification of products
2014/00607	Statement in an advertisement that could be an implication of a health benefit and would thus be subject to NHCR	Defining health claims
2015/00056	Concerned a claim that was used prior to the implementation of the NHCR	Excluded
2015/00276	Advertisement of a food supplement for which claims are used that are subject to the NHCR.	Defining health claims
2015/00526	Statement in an advertisement that could be an implication of a health benefit and would thus be subject to NHCR.	Defining health claims
2015/00916 - CVB	The use and substantiation of a health claim with a substance that is on the list of substances falling under the transitional measures	Transitional measures
2016/00348	Comments on the food information to consumer subject to Regulation (EC) No 1169/2011	Excluded
2016/00871	Use of medicinal claims and thus sales of medicinal products by presentation. No formal fines just breach of compliance of existing advertising code.<br><br>Use of testimonials with claims that are subject to the NHCR.	Classification of products<br><br>Commercial communication
2016/00873	Provision of information that are considered claims subject to the NHCR	Defining health claims

	Use of medicinal claims and thus sales of medicinal products by presentation. No formal fines just breach of compliance of existing advertising code.	Classification of products
2017/00539	Use of medicinal claims and thus sales of medicinal products by presentation. No formal fines just breach of compliance of existing advertising code.<br><br>A video presents misleading claims. The video was not made by producer but an affiliate.	Classification of products<br><br><br><br>Commercial communication
2017/00555	Use of medicinal claims and thus sales of medicinal products by presentation. No formal fines just breach of compliance of existing advertising code.	Classification of products
2017/00664	Use of medicinal claims and thus sales of medicinal products by presentation. No formal fines just breach of compliance of existing advertising code.	Classification of products
2017/00794	Use of medicinal claims and thus sales of medicinal products by presentation. No formal fines just breach of compliance of existing advertising code.	Classification of products
2018/00320/A	Use of health claims on substances that currently fall under the transitional measures. Specific review of evidence to substantiate the on-hold claims.	Transitional measures
2018/00338	Veterinary product	Excluded
2018/00562	Use of medicinal claims and thus sales of medicinal products by presentation. No formal fines just breach of compliance of existing advertising code.<br><br>Use of health claims on substances that currently fall under the transitional measures. Specific review of evidence to substantiate the on-hold claims.	Classification of product<br><br><br><br>Transitional measures
2019/00581	Veterinary product	Excluded
2020/00279	The reviews of the product on the retailer's website contained comments related to the treatment of disease. The reviews are considered responsibility of the retailer and the comments are thus subject to the NHCR.	Commercial communication
2020/00283	The FBO use health claims for food supplements. The claims are either authorised or on-hold. The claims falling under the transitional measures meet the general requirements: the disclaimer is used.	Transitional measures
2020/00311	Use of medicinal claims and thus sales of medicinal products by presentation. No formal fines just breach of compliance of existing advertising code.	Classification of products
2020/00509	Provision of information in an advertorial is subject to the NHCR	Commercial communication
2020/00510	The used wording of an authorised claim was challenged as it was believed to be deviating too much of the authorised claims and subsequent allowed translations	Defining health claims
2021/00346	Use of false and vague claims as well as unsubstantiated claims that are not falling under the transitional measures.	Defining health claims
2021/00562	Concerned the invitation for research send via e-mail	Excluded
2023/00086	Use of medicinal claims and thus sales of medicinal products by presentation. No formal fines just breach of compliance of existing advertising code.	Classification of products
2023/00130	Provision of information in an advertorial is subject to the NHCR	Commercial communication
2023/00169	Advertisement of a food supplement for which claims are used that are subject to the NHCR.	Defining health claims
2023/00470	Advertisement of a food supplement for which claims are used that are subject to the NHCR.	Defining health claims
